# Familial risk and heritability of diagnosed borderline personality disorder: a register study of the Swedish population

**DOI:** 10.1038/s41380-019-0442-0

**Published:** 2019-06-03

**Authors:** Charlotte Skoglund, Annika Tiger, Christian Rück, Predrag Petrovic, Philip Asherson, Clara Hellner, David Mataix-Cols, Ralf Kuja-Halkola

**Affiliations:** 1 Centre for Psychiatry Research, Department of Clinical Neuroscience, Karolinska Institutet, & Stockholm Health Care Services, Stockholm County Council, Stockholm, Sweden; 2 Department of Medical Epidemiology and Biostatistics, Karolinska Institutet, Stockholm, Sweden; 3 Department of Clinical Neuroscience, Karolinska Institutet, Stockholm, Sweden; 4 Social, Genetic and Developmental Psychiatry Centre, Institute of Psychiatry, Psychology, and Neuroscience, King’s College, London, UK

**Keywords:** Genetics, Psychiatric disorders

## Abstract

Family and twin studies of Borderline Personality Disorder (BPD) have found familial aggregation and genetic propensity for BPD, but estimates vary widely. Large-scale family studies of clinically diagnosed BPD are lacking. Therefore, we performed a total-population study estimating the familial aggregation and heritability of clinically diagnosed BPD. We followed 1,851,755 individuals born 1973–1993 in linked Swedish national registries. BPD-diagnosis was ascertained between 1997 and 2013, 11,665 received a BPD-diagnosis. We identified relatives and estimated sex and birth year adjusted hazard ratios, i.e., the rate of BPD-diagnoses in relatives to individuals with BPD-diagnosis compared to individuals with unaffected relatives, and used structural equation modeling to estimate heritability. The familial association decreased along with genetic relatedness. The hazard ratio was 11.5 (95% confidence interval (CI) = 1.6–83.8) for monozygotic twins; 7.4 (95% CI = 1.0–55.3) for dizygotic twins; 4.7 (95% CI = 3.9–5.6) for full siblings; 2.1 (95% CI = 1.5–3.0) for maternal half-siblings; 1.3 (95% CI = 0.9–2.1) for paternal half-siblings; 1.7 (95% CI = 1.4–2.0) for cousins whose parents were full siblings; 1.1 (95% CI = 0.7–1.8) for cousins whose parents were maternal half-siblings; and 1.9 (95% CI = 1.2–2.9) for cousins whose parents were paternal half-siblings. Heritability was estimated at 46% (95% CI = 39–53), and the remaining variance was explained by individually unique environmental factors. Our findings pave the way for further research into specific genetic variants, unique environmental factors implicated, and their interplay in risk for BPD.

## Introduction

Borderline personality disorder (BPD) is a complex psychiatric disorder characterized by emotional dysregulation [1, 2], severely impaired interpersonal functioning, and high risk of suicide [3]. Prevalence estimates range from 0.5 to 5.9% [4, 5] with a median of approximately 1.6% [6].

The causes of BPD are poorly understood [6] but it has been suggested to be associated with a low cognitive core capacity related to emotional regulation, built up by multiple sub-components that may be influenced both genetic and environmental causes [1]. BPD aggregates in families [7–16] but previous estimates of absolute risk in first-degree relatives range widely from 0.8% [15] to 18.3% [13]. Methodological differences, such as studying clinically diagnosed BPD versus dimensions of BPD-traits, or ascertaining case status by clinical interview, self-rating questionnaire or indirectly by informants, most likely explain much of the variability. Only one family study investigated clinically diagnosed BPD in both individuals and their relatives, and found an absolute risk of 14.1% in their first-degree relatives, corresponding to a 3.9 times increased risk compared to relatives of non-affected individuals [12]. However, the study population was at risk of selection bias and the study did not report on sex differences. The latter may be important, given the known female preponderance of the diagnosis [17]. Critically, previous family studies of BPD-diagnosis only included first-degree relatives, and could therefore not disentangle genetic from environmental contributions to the observed familiality.

Twin and extended twin studies, including relatives of twins, enable exploration of the relative contribution of genetic and environmental factors to familial aggregation [18]. Previous studies investigating the genetic impact of BPD show widely ranging heritability estimates, from 0 to 72% [19–37]. Among the population-based studies only seven ascertained case status by clinical interviews [20, 29, 31–33, 35, 37] narrowing down heritability to 32 to 72%. However, all these studies were based on sub-threshold BPD/BPD-traits and/or non-random selection of participants.

In summary, due to heterogeneous assessment procedures, questionable diagnostic validity, risk for biased estimates, and underpowered studies due to small sample sizes, there is considerable uncertainty regarding the familiality and heritability of BPD. Further, it is unclear how the results of many studies relate to clinically diagnosed BPD. To provide more precise and reliable estimates of familial clustering and heritability of BPD, we conducted a population-based familial aggregation study of more than 1.8 million individuals, including 11,665 individuals with clinically diagnosed BPD. We also applied quantitative genetic modeling in order to disentangle the genetic and environmental contributions. In addition, we examined possible sex differences in the patterns of familiality of BPD.

## Materials and methods

We performed a register linkage of the National Patient Register (NPR) [38], the Multi-Generation Register (MGR) [39], the Swedish Twin Registry (STR) [40], the Total Population Register [41], the Cause of Death Register [42], and the Medical Birth Register (MBR) [43]. The Regional Ethical Review Board in Stockholm, Sweden, provided ethical approval for the study (Dnr 2013/862–31/5). The requirement of informed consent was waived since no individual was contacted.

### Study population

We identified 2,181,047 individuals born between 1973 and 1993 through the MBR. Individuals born with congenital malformation (113,737), who died (9121) or emigrated (190,047) before their 15th birthday or 1 Jan 1997, and whose biological parents could not be identified (16,387), were excluded. In all, our analytical sample comprised 1,851,755 individuals.

Using the MGR and the STR we identified all pairs of following relatives types (degree of genetic relatedness, i.e., the average proportion of co-segregating alleles shared between relatives, within brackets) monozygotic (MZ) twins (100%), dizygotic (DZ) twins (50%), full siblings (50%), maternal half-siblings (25%), paternal half-siblings (25%), cousins whose parents were full siblings (12.5%), and cousins whose parents were maternal half-siblings and paternal half-siblings (6.25%). We paired every individual with each relative of the different types that were identifiable, the relative indexing the exposure and the individual the outcome. Thus, each relative pair was included twice, once with an individual as exposure person and once as outcome person.

### Study variables

#### Clinical diagnosis of BPD

We identified individuals with BPD using first date of International Classification of Diseases 10th revision (ICD-10) diagnoses for Emotionally Unstable Personality Disorder (ICD-10 code F60.3) after their 15^th^ birthday, in the NPR from 1997 (when ICD-10 was introduced in Sweden). NPR includes inpatient data throughout, and outpatient data from 2001. Our linkage was updated through 2013. In the Swedish version of the ICD-10, the F60.3 diagnosis code corresponds to the DSM-IV-TR criteria for BPD [44]. We have previously performed a medical chart validation study, showing that 81% of investigated medical charts with F60.3 diagnosis codes appropriately captured the DSM-IV-TR BPD-diagnosis [45].

#### Covariates

To adjust for cohort effects, birth year was included as a potential confounder and treated as a categorical variable (1973–1977, 1978–1982, 1983–1987, 1988–1993). Furthermore, to account for potential incidence and prevalence differences between men and women, sex was included in the analyses as a covariate.

#### Comorbidities

In addition, we identified ICD-10 diagnoses of psychiatric disorders and self-harm from the NPR clustered into broad groups (Table 1). The follow-up period was identical with that for BPD and the diagnoses were treated as dichotomous variables (i.e., present or absent), regardless of when they were recorded.

**Table 1:** Descriptive, number of individuals (column percent)

	Not BPD	BPD	ICD-10 codes
*N* (row percent)	1,840,090 (99.4)	11,665 (0.6)	NA
Sex			
Male	953,487 (51.8)	1699 (14.6)	NA
Female	886,603 (48.2)	9966 (85.4)	NA
Birth year			
1973–1977	419,606 (22.8)	2208 (18.9)	NA
1978–1982	385,725 (21.0)	2826 (24.2)	NA
1983–1987	410,953 (22.3)	3343 (28.7)	NA
1988–1993	623,806 (33.9)	3288 (28.2)	NA
Having a half-sibling	534,361 (29.0)	5543 (47.5)	NA
Comorbidities^a^			
Anxiety disorders	107,264 (5.8)	8836 (75.7)	F40-F42, F43.1
Affective disorders	107,169 (5.8)	8864 (76.0)	F30-F39
Substance use disorders	81,583 (4.4)	5672 (48.6)	F10-F16, F18-F19
Psychotic disorders	11,526 (0.6)	1401 (12.0)	F20-F29
Neurodevelopmental disorders	57,116 (3.1)	3625 (31.1)	F70-F79, F84, F90, F95
Conduct disorders	3172 (0.2)	317 (2.7)	F91
Eating disorders	17,188 (0.9)	2114 (18.1)	F50
Personality disorders	15,898 (0.9)	4856 (41.6)	F60-F69, excluding F60.3
Any of above psychiatric disorders	207,861 (11.3)	11,169 (95.7)	Any of above
Self-harm	55,583 (3.0)	6063 (52.0)	X60-X84, Y10-Y34

*NA* not applicable. All variables are statistically significantly different between Not BPD and BPD group according to Pearson *χ*^2^-tests, with *p*-values < 0.001

^a^In Supplemental eTable 4 the included disorder codes are explained in more detail

### Statistical analyses

#### Descriptive

Number of individuals with and without a BPD-diagnosis were calculated per sex, categorized birth year, half-sibling status, and included comorbid disorders. We estimated cumulative incidence for the full cohort, stratified per sex and birth year, using a Kaplan-Meier approach.

#### Familial aggregation

Risk of BPD was estimated through Cox proportional hazards regression, adjusted for sex and birth year of individual and relative. We censored individuals at emigration, death, or end of follow-up, whichever came first. We accounted for time during which a diagnosis was not observable (i.e., prior to 1997 or age 15) by allowing delayed entry into the at-risk group in analyses. Informed by previous studies [46, 47] we used a time-varying exposure approach; individuals were unexposed until the date of a relative’s diagnosis, and exposed thereafter. Additionally, we performed the analysis treating BPD as a binary variable, ignoring time of follow-up, in logistic regressions. Finally, again viewing BPD as a binary variable, we estimated concordance rates (the proportion of relatives of an individual with BPD also having BPD) and tetrachoric correlations.

Confidence intervals were obtained using a cluster robust sandwich estimator to adjust for non-independence between relative pairs.

#### Quantitative genetic analysis

We used a quantitative genetic approach similar to previous studies [46, 47], which is based on classical twin methodology [48]. Briefly, from each family we included only the sibling pair with the shortest time between births from each nuclear family of MZ twins, DZ twins, full siblings, maternal and paternal half-siblings. We used the liability-threshold model, where an underlying normally distributed liability of disease is assumed. If an individual has the diagnosis, the risk-liability is assumed to be over an estimated threshold. The inferred correlation between liabilities in relatives is equivalent to a tetrachoric correlation, and the basis for the quantitative genetic model. We estimated the additive genetic effects (A), i.e., how different alleles of various loci affect the phenotypic outcome independently and additively, also known as narrow sense heritability, on the variance of the disorder. Additive genetic factors were assumed shared 100% between MZ twins (as they are genetically identical), 50% between full siblings and DZ twins, and 25% between half-siblings. Further, we estimated dominance deviations (D), the deviation from additive genetic effects due to interactions within genetic loci. Dominance effects were assumed shared 100% between MZ twins, 25% between full siblings and DZ twins, and 0% between half-siblings. We also calculated the broad-sense heritability (A + D), reflecting the total variance due to genetic influences. We estimated shared family environmental effects (C), i.e., environmental influences that have the effect of making siblings more similar to one another, such as socioeconomic status, religious beliefs in the family, or parental rearing style. Similar to previous studies [46, 47], we assumed that C was shared to an equal extent in all siblings except paternal half-siblings. Finally, we estimated individually unique environmental effects (E), which are not shared between relatives, such as experiencing a medical condition or sexual abuse. We fitted the full model, referred to as the ADCE-model, and compared the goodness of fit of reduced models (ACE, ADE, DCE, AE, CE, DE, and E), using likelihood ratio tests. We also used Akaike’s Information Criterion (AIC) for model comparisons, which favors model parsimony and allows for comparison across non-nested models. We adjusted the prevalence for sex, birth year (in categories), and being a half-sibling.

We used the statistical software R and packages survival [49], drgee [50], polycor [51], and OpenMx [52] for analyses.

#### Sensitivity analyses

For full siblings, we performed the familial aggregation analyses separately for sex-combinations of exposure and outcome individuals, as well as separately per birth year categories.

Among females only, we calculated concordance rates and tetrachoric correlations for female siblings in a subsample identified equivalently as outlined above. Finally, we performed quantitative genetic analysis on females only using the same subsample.

## Results

### Descriptive

Table 1 shows that more females than males were diagnosed with BPD (1.1% versus 0.2%). BPD-diagnosis was less common in the oldest and youngest birth cohorts. The amount of comorbid disorders was high: 95.7% of individuals with BPD-diagnosis had at least one of the other included psychiatric diagnoses, compared to 11.3% in non-BPD individuals, and all included disorder groups were more prevalent among individuals with BPD. The most common comorbidities were anxiety disorders (75.7%), affective disorders (76.0%), and substance use disorders (48.6%). We have previously reported that 30.9% of individuals with BPD-diagnosis also has a diagnosis of Attention-Deficit/Hyperactivity Disorder [45]; in the current study 31.1% of BPD-diagnosed also had a neurodevelopmental disorder diagnosis. Self-harm diagnoses were common in BPD (52.0%), indicating that approximately half of those with BPD presented self-mutilating behaviors to the extent that they had received medical attention for their self-harm.

**Fig. 1:**
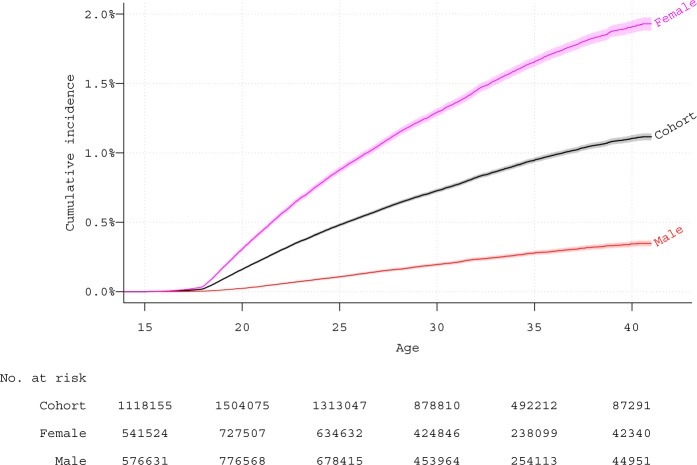
Cumulative incidence in full cohort and sub-cohorts of females and males. Kaplan-Meier estimates with 95% confidence intervals. Note: Cohort, full cohort. Female, all females in cohort. Male, all males in cohort

The cumulative incidence of BPD was approximately 0.5% (0.8% in females and 0.2% in males) by age 25 and increased to over 1% (almost 2% in females, and about 0.4% in males) by age 40 (Fig. 1). However, following further stratification by birth categories (Supplemental eFigure 1), the importance of calendar periods of follow-up became evident; showing the steepest increase in the youngest cohort, were follow-up in younger years was conducted in the most recent time period.

### Familial aggregation

**Fig. 2:**
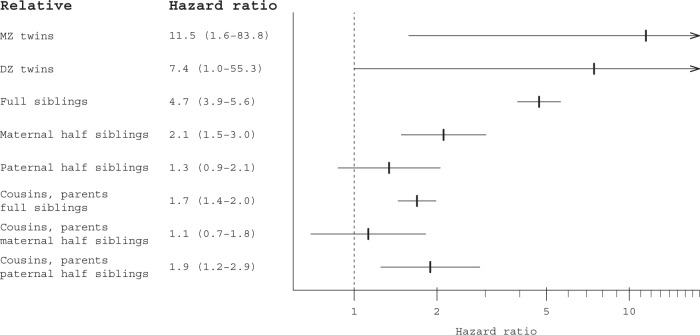
Familial aggregation. Hazard ratios (95% confidence interval). Note: X-axis uses logarithmic scale; plot with non-logarithmic scale can be found in Supplemental eFigure 2

Results of familial aggregation calculated through Cox regression are presented in Fig. 2 (Supplemental eTable 1 presents crude associations, and number of individuals and pairs included in analyses). Overall, the associations decreased along with decreasing genetic relatedness; MZ twins had an adjusted hazard ratio (HR) of 11.5 (95% confidence interval (CI) = 1.6–83.8), while the HR for DZ twins was 7.4 (95% CI = 1.0–55.3), and for full siblings 4.7 (95% CI = 3.9–5.6). For maternal half-siblings the HR was 2.1 (95% CI = 1.5–3.0), and for paternal half-siblings 1.3 (95% CI = 0.9–2.1) – a difference that was not statistically significant (*p*-value = 0.108). Cousins whose parents were full siblings had a HR of 1.7 (95% CI = 1.4–2.0), for cousins whose parents were maternal half-siblings it was 1.1 (95% CI = 0.7–1.8); and, for cousins whose parents were paternal half-siblings the HR was 1.9 (95% CI = 1.2–2.9).

In Supplemental eTable 2 results from the logistic regression analyses not taking follow-up time into account are presented. The results adjusted for birth period did not differ substantially from the main Cox-analyses.

Concordance rates and tetrachoric correlations in subsampled pairs of siblings are presented in Table 2. Very few twin pairs were concordantly diagnosed with BPD. The concordance rate for full siblings was 2.5% (95% CI = 1.9–3.0), a 5-fold increase compared to the baseline proportion of 0.5% among full siblings (see Table 1). Half-siblings had similar concordance rates as full siblings, however their baseline rate of diagnoses was higher (1.0%, Table 1), and thus the concordance rate correspond to a 2 to 3-fold increase. The tetrachoric correlations decreased with decreased genetic relatedness, from 0.45 in MZ twins to 0.08 in paternal half-siblings.

**Table 2:** Concordances and tetrachoric correlations (95% confidence interval)

	Concordant without BPD	Discordant BPD	Concordant BPD	Concordance rate^a^	Tetrachoric correlation
MZ twins	3130	25	1	7.4% (−6.3–21.1)	0.45 (0.07–0.83)
DZ twins	3987	46	1	4.2% (−3.7–12.1)	0.30 (−0.06–0.66)
Full siblings	526,571	5936	76	2.5% (1.9–3.0)	0.21 (0.18–0.25)
Maternal half-siblings	63,313	1615	22	2.7% (1.6–3.7)	0.12 (0.05–0.19)
Paternal half-siblings	61,045	1545	16	2.0% (1.1–3.0)	0.08 (−0.01–0.16)

Data consists of the pairs born closest to each other, or one random pair if several born similarly close

^a^Proportion of individuals with BPD-diagnosis whose relative also has BPD-diagnosis

### Quantitative genetic analysis

In Table 3 model fitting results and estimates from quantitative genetic analysis are presented. Compared to the ADCE-model, reduced models yielded statistically non-significant decrease of model fit for ACE, ADE, DCE, and AE-models, and the AIC favored the AE model. In the AE-model the heritability (A) was estimated at 46% (95% CI = 39–53), and non-shared environment (E) at 54% (95% CI = 47–61).

**Table 3:** Quantitative genetic analysis. Fitted sub-models compared to full ADCE model

	Model comparison measures					Explained variance, percent (95% confidence interval)				
Models	No. parameters	Akaike’s information criterion^a^	−2 log likelihood	Difference in −2 log likelihood^b^	*p*-value^b^	Additive genetic	Dominance genetic	Shared environment	Non-shared environment	Total genetic (broad sense heritability)
ADCE	9	−2,564,587.58	104,710.42	NA	NA	29 (0–52)	18 (0–59)	4 (0–16)	49 (27–69)	47 (18–73)
ACE	8	−2,564,588.84	104,711.16	0.74	0.388	39 (16–53)	NA	3 (0–14)	57 (47–71)	39 (16–53)
ADE	8	−2,564,589.02	104,710.98	0.56	0.454	40 (19–53)	14 (0–52)	NA	47 (25–61)	53 (39–75)
DCE	8	−2,564,586.96	104,713.04	2.62	0.106	NA	44 (15–69)	12 (6–18)	44 (24–67)	44 (15–69)
AE	7	−2,564,590.49	104,711.51	1.09	0.581	46 (39–53)	NA	NA	54 (47–61)	46 (39–53)
CE	7	−2,564,580.14	104,721.86	11.45	0.003	NA	NA	21 (17–24)	79 (76–83)	NA
DE	7	−2,564,574.55	104,727.45	17.03	< 0.001	NA	80 (68–89)	NA	20 (11–32)	80 (68–89)
E	6	−2,564,463.52	104,840.48	130.06	< 0.001	NA	NA	NA	100 (NA)	NA

*No. parameters* number of estimated parameters in model, *NA* not applicable. Analysis based on sub-sample presented in Table 2

^a^Lower value indicates better fit

^b^Difference in −2 log likelihood, and *p*-value for likelihood ratio test thereof, between model and ADCE model

### Sensitivity analyses

In the analyses of familial aggregation among full siblings, when one sex acted as both exposure and outcome individuals, males had a higher HR than females (11.6 versus 4.4, *p*-value = 0.020; Supplemental eTable 3). However, the support for sex differences did not permeate the analyses; none of the other comparisons between exposure-outcome combinations of sex was statistically significant. For birth years, the association was strongest for the oldest cohort, and weakest for the cohort born 1983–1987 (Supplemental eTable 3).

Among females, sibling concordance rates were higher, reflecting the higher prevalence of the disorder among women, but tetrachoric correlations did not differ considerably from the main estimates (Supplemental eTable 5). Consequently, quantitative genetic analysis on females only yielded very similar result as the main analysis, with an estimate of heritability of 47% (95% CI, 38–55) in the AE-model (Supplemental eTable 6).

## Discussion

This is the first total-population study of familial aggregation and heritability of clinically diagnosed BPD. Confirming previous studies, relatives of individuals with a diagnosis of BPD had a higher risk of receiving a BPD-diagnosis than those without familial vulnerability. For example, there was a 4.7 times increased risk for full siblings. Heritability of clinically diagnosed BPD was estimated at 46% (95% CI = 39–53), consistent with previous studies demonstrating the heritability of dimensional BPD-traits in general population twin samples. The best fitting model indicated little or no role for shared familial environmental factors. Thus, the pattern of familial aggregation of BPD across different types of relatives indicates that genetic factors play a significant role in the risk of developing BPD, and explain the familial clustering of the disorder.

Previous heritability estimates vary widely [19–37] and our results represent a substantial improvement in precision, based on our sample size and the number of BPD-diagnosed individuals, as reflected in the narrow confidence intervals for the heritability estimates. Furthermore, our findings indicate that close family members of individuals represent an important high-risk group for developing BPD; and that this is due to genetic, and not environmental, influences. This has important implications. Firstly, clinicians need to be aware of the elevated risks for BPD in relatives of BPD-patients. Secondly, although thus far no single nucleotide polymorphism (genetic variant) have been identified that reach genome-wide significance [53, 54], future molecular genetic studies may, given sufficient sample sizes, identify some of the genetic risk factors that confer risk for BPD.

The results also estimate a 54% contribution of non-shared, individually unique, environmental factors. Shared environment did not influence the statistical goodness of fit, suggesting that shared familial environmental factors, such as socioeconomics, are unlikely to contribute substantially to the etiological underpinnings of BPD. Traumatic life events, such as sexual or physical abuse and parental divorce or illness, are more frequently reported by individuals with BPD compared to healthy controls or patients with other personality disorders [8, 55–58]. Although no given environmental risk factor has yet to be clearly identified as causative, our findings suggest that these may reflect unique environmental risks to individuals within a family or might act via gene-by-environment interactions. Further, these environmental associations could also reflect gene-environment correlations, additionally complicating the identification of specific environmental risks. The identification of unique environmental risk factors and gene-by-environment interactions for BPD should be regarded a research priority, as these are potentially preventable or modifiable via early interventions.

In line with previous research, the pattern of comorbid disorders showed that comorbidity is a hallmark of BPD. One potential cause for high comorbidity rates is genetic overlap since recent research findings indicate high genetic overlap across many psychiatric disorders and a general genetic propensity [59, 60]. Thus, our results are likely to partly reflect non-specific genetic effects that acts across mental health disorders. Here we did not adjust our analyses for any comorbid conditions since adjustment would most likely influence our results in a non-interpretable way.

On a mechanistic level, it has been suggested that several underlying neural and cognitive processes involved in emotional regulation are dysfunctional in BPD [1]. The present study suggests that that these underlying deficits are largely genetically predetermined. Such regulatory processes are thought to involve widely distributed large-scale neural networks, including specific prefrontal and anterior cingulate regions, networks involved in emotional processing, basal ganglia circuits, and neuromodulatory systems such as dopamine and noradrenaline systems [1, 2].

Since we had categorical yes/no measure of BPD, we necessarily treated it as a categorical variable in analyses, rather than having continuous measures or separate symptoms. Concurrent research has found that many psychiatric diagnoses are representable as the extreme end of a continuous trait [61], although no study specifically addressing this in BPD has been performed. Further, recent research supports a close association between categorical BPD-criteria (i.e., BPD-symptoms) and corresponding trait dimensions of personality disorders [62, 63]. Thus, BPD-diagnoses, as used in current study, likely represent the same underlying psychopathological construct as previous work using symptoms and/or dimensional measures of BPD-traits.

This study has several important strengths including utilizing Swedish nationwide register linkages. Previous studies are often limited by small sample sizes [7–10, 13–16, 19, 34, 36] the use of BPD-traits rather than diagnosis [7, 9, 10, 19–24, 28, 29, 31–33, 35–37, 64], and self-rating questionnaires [16, 19, 21–28, 30] rather than clinical diagnoses, exposing them to risks of low statistical power, selection- and recall bias. This register-based population cohort provided large sample-size, well-identified biological relatives, extensive follow-up time, and clinical diagnoses. Furthermore, data was gathered consecutively and independently from the current study. Moreover, previous studies investigating etiological underpinnings of BPD are predominantly based on twins. This study uses multiple types of relatives, and quantitative genetic analysis was performed in a sibling sample.

Our results need to be interpreted in light of some limitations. First, we most likely underestimate the true proportion of individuals with symptom levels and impairments corresponding to BPD-diagnosis (i.e., more cases would be identified if everyone in the cohort was assessed for BPD). Second, it is possible that we are missing male individuals with BPD in our population sample as the female to male ratio in our study is 5.5:1, whereas a more even sex ratio has been reported in a US community sample assessed by interview [4]. Results remained stable in women, but we did not have sufficient power to perform the quantitative genetic analysis in men; thus, generalization of our findings to men with BPD should be made with caution. Third, the BPD-diagnosis is based on fulfilling at least five out of nine symptom criteria leading to clinically significant distress or functional impairment in important life areas, and thus a substantial heterogeneity of symptom profiles exists. Although heterogeneity may hamper specificity of the diagnosis, our previous medical chart validation study demonstrated the correspondence of the register-based diagnoses with the DSM-IV criteria [45]. Further, identified individuals with a diagnosis of BPD in our population comprise those in contact with the health care system, thus representing the “real world” cases, and are most likely a representative sample of individuals suffering from functional impairment related to BPD-symptoms. Fourth, our methods will not detect individuals diagnosed in outpatient care prior to 2001. Importantly, however, under-detection of BPD is likely to bias the estimates towards the null. Fifth, relatives to individuals with a BPD-diagnosis may be in closer contact with health care and consequently be more likely to receive a diagnosis, thus inflating the estimates due to detection bias. Sixth, BPD is reported to be most prevalent in adolescence, and its prevalence decreases significantly towards the age of 24 [19]. We detected different incidence rates of BPD across different calendar periods with an increased propensity of assigning BPD-diagnosis at earlier ages, a phenomenon that may indicate changes in diagnostic practice. The extended twin/family design makes it less sensitive to trends in diagnostic practice; if time or local trends are present they are likely to apply to the population in a similar way. Lastly, our follow-up periods started later in older cohorts. The results might be affected by left truncation, missing earlier onset BPD. However, BPD is often described as a disorder of “stable instability” [6, 23, 55, 65, 66], resulting in repeated interactions with health care, increasing the likelihood of being identified as carrying a diagnosis in the current study.

In conclusion, BPD aggregates in families and the heritability was estimated at 46%, with the remaining variance explained by non-shared environmental factors. This finding is important for further expanding our understanding of BPD. The time is ripe for identification of genetic variants associated with BPD through large scale genome-wide studies, for identification of environmental risk factors, and of how these correlate or interact to increase the risk of BPD.

## Supplementary information

Supplemental online material
