# Supplementary material for: Familial risk and heritability of diagnosed borderline personality disorder: a register study of the Swedish population
Source: Mol Psychiatry. 2019 Jun 3;26(3):999–1008. doi: 10.1038/s41380-019-0442-0 (PMC7910208; doi:10.1038/s41380-019-0442-0)
Supplement: Supplementary file 1 — Supplemental online material [file 41380_2019_442_MOESM1_ESM.docx]

**Familial Risk and Heritability of Diagnosed Borderline Personality Disorder:
A Register Study of the Swedish Population**

**Supplemental online material**

Charlotte Skoglund, MD, PhD^1^; Annika Tiger, MD^2^; Christian Rück, MD, PhD^1^; Predrag Petrovic, MD, PhD^3^; Philip Asherson, MD, PhD^4^; Clara Hellner, MD, PhD^1^; David Mataix-Cols, PhD^1^; Ralf Kuja-Halkola, PhD^2^

**These authors contributed equally:** Charlotte Skoglund and Annika Tiger.

**Author Affiliations:**^1^ Centre for Psychiatry Research, Department of Clinical Neuroscience, Karolinska Institutet, & Stockholm Health Care Services, Stockholm County Council.
^2^ Department of Medical Epidemiology and Biostatistics, Karolinska Institutet, Stockholm, Sweden.
^3^ Department of Clinical Neuroscience, Karolinska Institutet, Stockholm, Sweden.
^4^ Social, Genetic and Developmental Psychiatry Centre, Institute of Psychiatry, Psychology, and Neuroscience, King's College, London, UK.

**Corresponding author**: Ralf Kuja-Halkola, Karolinska Institutet, Department of Medical Epidemiology and Biostatistics, Karolinska Institutet, PO Box 281, SE-171 77 Stockholm, Sweden. E-mail: [Ralf.Kuja-Halkola@ki.se](mailto:Ralf.Kuja-Halkola@ki.se)

**Supplemental eTable 1.** Familial aggregation analyses, number of pairs and hazard ratios (95% confidence interval).

|  | **No. individuals**^a^ | **No. analytic pairs**^b^ | **No. unique pairs** | **No. families**^c^ | **Crude** | **Adjusted**^d^ |
| --- | --- | --- | --- | --- | --- | --- |
| **MZ twins** | 6314 | 6314 | 3157 | 3156 | 19.1 (3.0–122.1) | 11.5 (1.6–83.8) |
| **DZ twins** | 8076 | 8076 | 4038 | 4034 | 6.8 (0.9–53.9) | 7.4 (1.0–55.3) |
| **Full siblings** | 1,236,459 | 1,787,292 | 893,646 | 532,583 | 5.5 (4.6–6.5) | 4.7 (3.9–5.6) |
| **Maternal half-siblings** | 173,954 | 241,170 | 120,585 | 64,950 | 2.5 (1.8–3.6) | 2.1 (1.5–3.0) |
| **Paternal half-siblings** | 175,046 | 252,086 | 126,043 | 62,606 | 1.7 (1.1–2.5) | 1.3 (0.9–2.1) |
| **Cousins, parents full siblings** | 1,369,037 | 5,567,034 | 2,783,517 | 434,408 | 2.0 (1.7–2.4) | 1.7 (1.4–2.0) |
| **Cousins, parents maternal half-siblings** | 139,844 | 338,040 | 169,020 | 30,034 | 1.4 (0.9–2.2) | 1.1 (0.7–1.8) |
| **Cousins, parents paternal half-siblings** | 143,111 | 320,618 | 160,309 | 30,481 | 2.2 (1.5–3.3) | 1.9 (1.2–2.9) |

^a^ Total number of unique individuals in analysis.

^b^ Total number of ways relatives are paired. Every pair is included twice; with relative A as exposure and relative B as outcome, and vice versa.

^c^ Number of families relatives are identified from. For siblings indexed by mother-father combinations (MZ, DZ, and full siblings), or by mothers (maternal half-siblings) or fathers (paternal half-siblings). For cousins indexed by grandmother-grandfather combinations (cousins whose parents are full siblings), or by grandmothers (cousins whose parents are maternal half-siblings) or grandfathers (cousins whose parents are paternal half-siblings).

^d^ Adjusted for sex and birth year in categories of index individual and relative.

**Supplemental eTable 2.** Familial aggregation using binary measures of disease. Odds ratios (95% confidence interval).

|  | **Crude** | **Adjusted**^a^ |
| --- | --- | --- |
| **MZ twins** | 20.0 (2.4–165.5) | 14.5 (1.7–124.6) |
| **DZ twins** | 7.5 (1.0–58.2) | 7.9 (0.9–68.3) |
| **Full siblings** | 4.7 (3.9–5.6) | 4.8 (4.0–5.7) |
| **Maternal half-siblings** | 2.3 (1.7–3.2) | 2.2 (1.6–3.2) |
| **Paternal half-siblings** | 1.2 (0.8–1.8) | 1.2 (0.8–1.8) |
| **Cousins, parents full siblings** | 1.7 (1.5–2.0) | 1.7 (1.5–2.0) |
| **Cousins, parents maternal half-siblings** | 1.3 (0.8–1.9) | 1.3 (0.8–1.9) |
| **Cousins, parents paternal half-siblings** | 1.7 (1.1–2.5) | 1.7 (1.2–2.6) |

^a^ Adjusted for sex and birth year in categories of index individual and relative.

**Supplemental eTable 3.** Stratified analyses of familial aggregation among full siblings. Hazard ratio (95% confidence interval).

| **Full siblings** | **Crude** | **Adjusted**^a^ |
| --- | --- | --- |
| **Sex**^b^ |  |  |
| Male exposure, male outcome | 12.7 (5.8–28.0) | 11.6 (5.3–25.4) |
| Female exposure, female outcome | 5.2 (4.2–6.5) | 4.4 (3.5–5.5) |
| Male exposure, female outcome | 5.6 (3.4–9.1) | 4.9 (3.0–8.0) |
| Female exposure, male outcome | 5.6 (3.5–9.1) | 5.3 (3.3–8.5) |
| **Birth year** |  |  |
| 1973–1977 | 8.4 (4.6–15.3) | 7.8 (4.3–14.3) |
| 1978–1982 | 6.0 (3.6–10.0) | 5.8 (3.5–9.8) |
| 1983–1987 | 2.1 (1.1–4.2) | 2.2 (1.1–4.5) |
| 1988–1993 | 5.3 (3.3–8.4) | 5.8 (3.6–9.2) |

^a^ Adjusted for sex and birth year in categories of index individual and relative, where applicable.

^b^ P-values for differ sex-comparisons for the adjusted estimates:

|  | Female exposure, female outcome | Male exposure, female outcome | Female exposure, male outcome |
| --- | --- | --- | --- |
| Male exposure, male outcome | 0.020 | 0.072 | 0.072 |
| Female exposure, female outcome | NA | 0.677 | 0.494 |
| Male exposure, female outcome | 0.677 | NA | 0.842 |

**Supplemental eTable 4.** ICD-codes with explanations for comorbidities included in **Table 1**.

| **Disorder group** | **ICD-codes** | **Explanation** |
| --- | --- | --- |
| **Anxiety disorders** | F40 | Phobic anxiety disorder. |
|  | F41 | Anxiety disorders. |
|  | F42 | Obsessive-compulsive disorder. |
|  | F43.1 | Post-traumatic stress disorder. |
| **Affective disorders** | F30-F31 | Bipolar spectrum disorder. |
|  | F32-F39 | Depressive and affective disorders. |
| **Substance use disorders** | F10-F19, excluding F17 | Mental and behavioural disorders due to psychoactive substance use. Excluded diagnosis, F17, pertains to tobacco. |
| **Psychotic disorders** | F20 | Schizophrenia disorder. |
|  | F21 | Schizotypal disorder. |
|  | F22-F29 | Persistent and transient delusional and psychotic disorders. |
| **Neurodevelopmental disorders** | F70-F79 | Mental retardation. |
|  | F84 | Autism spectrum disorder. |
|  | F90 | Attention deficit/hyperactivity disorder. |
|  | F95 | Tic disorders, including Tourette disorder. |
| **Conduct disorders** | F91 | Conduct disorders. |
| **Eating disorders** | F50 | Anorexia nervosa, bulimia nervosa, overeating, vomiting, and other eating disorders. |
| **Personality disorders** | F60-F69, excluding F60.3 | Disorders of adult personality and behavior. |
| **Self-harm** | X60-X84 | Intentional self-harm. |
|  | Y10-Y34 | Event of undetermined intent. |

**Supplemental eTable 5.** Females only. Concordances and tetrachoric correlations (95% confidence interval).

|  | Concordant without BPD | Discordant BPD | Concordant BPD | Concordance rate^a^ | Tetrachoric correlation |
| --- | --- | --- | --- | --- | --- |
| **MZ twins** | 1838 | 22 | 1 | 8.3% (-7.0–23.6) | 0.44 (0.03–0.84) |
| **DZ twins** | 1109 | 17 | 1 | 10.5% (-8.5–29.6) | 0.47 (0.03–0.90) |
| **Full siblings** | 155,015 | 3088 | 64 | 4.0% (3.0–4.9) | 0.23 (0.18–0.28) |
| **Maternal half-siblings** | 20,123 | 924 | 26 | 5.3% (3.4–7.3) | 0.17 (0.09–0.25) |
| **Paternal half-siblings** | 20,172 | 907 | 14 | 3.0% (1.5–4.5) | 0.06 (-0.04–0.15) |

Note: Data consists of the pairs born closest to each other, or one random pair if several born similarly close.

^a^ Proportion of individuals with BPD diagnosis whose relative also has BPD diagnosis.

**Supplemental eTable 6.** Females only. Quantitative genetic analysis. Fitted sub-models compared to full ADCE model.

|  | Model comparison measures | | | | | Explained variance, percent (95% confidence interval) | | | | |
| --- | --- | --- | --- | --- | --- | --- | --- | --- | --- | --- |
| Models | No. parameters | Akaike’s information criterion^a^ | -2 log likelihood | Difference in -2 log likelihood^b^ | P-value^b^ | Additive genetic | Dominance genetic | Shared environment | Non-shared environment | Total genetic (broad sense heritability) |
| ADCE | 8 | -758,834.09 | 54,433.91 | NA | NA | 20 (0–51) | 11 (0–56) | 11 (0–22) | 58 (32–78) | 32 (1–63) |
| ACE^c^ | 7 | -758,835.88 | 54,434.12 | 0.21 | 0.648 | 27 (0–52) | NA | 10 (0–22) | 63 (48–79) | 27 (0–52) |
| ADE | 7 | -758,833.36 | 54,436.64 | 2.73 | 0.099 | 46 (24–55) | 2 (0–44) | NA | 52 (28–62) | 48 (38–72) |
| DCE | 7 | -758,835.09 | 54,434.91 | 1.00 | 0.317 | NA | 30 (0–60) | 16 (9–24) | 54 (30–80) | 30 (0–60) |
| AE^c^ | 6 | -758,835.35 | 54,436.65 | 2.73 | 0.255 | 47 (38–55) | NA | NA | 53 (45–62) | 47 (38–55) |
| CE | 6 | -758,833.93 | 54,438.07 | 4.15 | 0.125 | NA | NA | 22 (18–26) | 78 (74–82) | NA |
| DE | 6 | -758,817.36 | 54,454.64 | 20.72 | <0.001 | NA | 78 (65–89) | NA | 22 (11–35) | 78 (65–89) |
| E | 5 | -758,735.08 | 54,538.92 | 105.01 | <0.001 | NA | NA | NA | 100 (NA) | NA |

Note: No. parameters, number of estimated parameters in model. NA, not applicable. Analysis based on sub-sample presented in **eTable 5**.

^a^ Lower value indicates better fit.

^b^ Difference in -2 log likelihood, and p-value for likelihood ratio test thereof, between model and ADCE model.

^c^ A likelihood ratio test between the ACE- and AE-models yielded a χ^2^ test-statistic of 2.52 and, with one degree of freedom, a p-value of 0.112.

**Supplemental eFigure 1.** Cumulative incidence in sub-cohorts of females and males born different periods. Kaplan-Meier estimates with 95% confidence intervals.


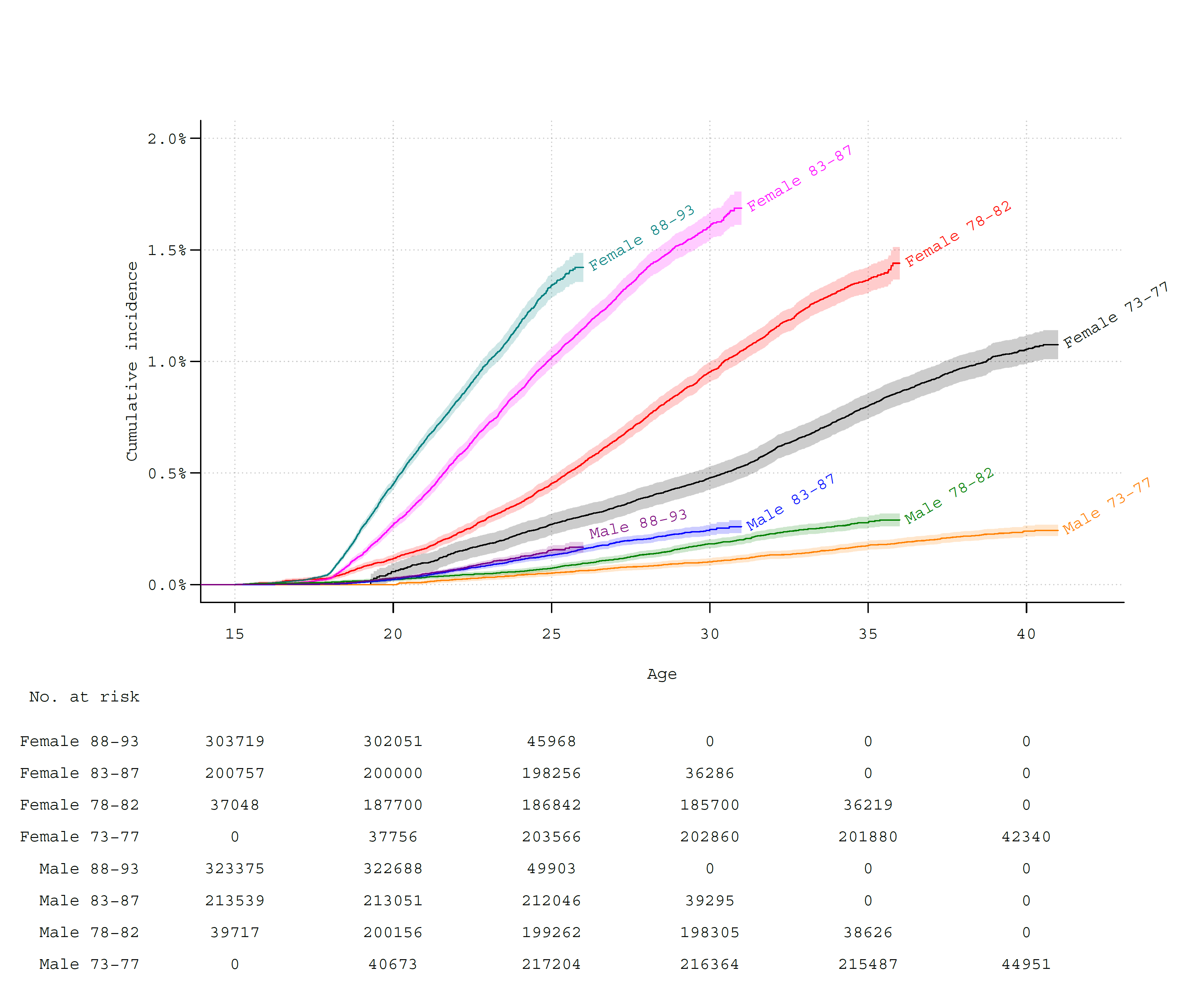
Note: 73-77, sub-cohort born 1973-1977. 78-82, sub-cohort born 1978-1982. 83-87, sub-cohort born 1983-1987. 88-93, sub-cohort born 1988-1993.

**Supplemental eFigure 2.** Familial aggregation. Hazard ratios (95% confidence intervals).

Note: X-axis is not on logarithmic scale.
